# Lipidomic Analyses Uncover Apoptotic and Inhibitory Effects of Pyrvinium Pamoate on Cholangiocarcinoma Cells via Mitochondrial Membrane Potential Dysfunction

**DOI:** 10.3389/fpubh.2021.766455

**Published:** 2021-12-07

**Authors:** Yingpinyapat Kittirat, Jutarop Phetcharaburanin, Bundit Promraksa, Thanaporn Kulthawatsiri, Arporn Wangwiwatsin, Poramate Klanrit, Sakkarn Sangkhamanon, Apiwat Jarearnrat, Suyanee Thongchot, Panupong Mahalapbutr, Watcharin Loilome, Hideyuki Saya, Nisana Namwat

**Affiliations:** ^1^Department of Biochemistry, Faculty of Medicine, Khon Kaen University, Khon Kaen, Thailand; ^2^Faculty of Medicine, Cholangiocarcinoma Research Institute, Khon Kaen University, Khon Kaen, Thailand; ^3^Khon Kaen University International Phenome Laboratory, Khon Kaen University Science Park, Innovation and Enterprise Affairs, Khon Kaen University, Khon Kaen, Thailand; ^4^Faculty of Medical Technology, Nakhonratsima College, Nakhon Ratchasima, Thailand; ^5^Department of Pathology, Faculty of Medicine, Khon Kaen University, Khon Kaen, Thailand; ^6^Department of Surgery, Faculty of Medicine, Khon Kaen University, Khon Kaen, Thailand; ^7^Department of Immunology, Faculty of Medicine Siriraj Hospital, Mahidol University, Bangkok, Thailand; ^8^Research Department, Faculty of Medicine Siriraj Hospital, Siriraj Center of Research Excellence for Cancer Immunotherapy (SiCORE-CIT), Mahidol University, Bangkok, Thailand; ^9^Division of Gene Regulation, Institute for Advanced Medical Research, School of Medicine, Keio University, Tokyo, Japan

**Keywords:** pyrvinium pamoate, cholangiocarcinoma, lipidomic, apoptosis, mitochondrial membrane potential dysfunction

## Abstract

Pyrvinium pamoate (PP), an FDA-approved anthelmintic drug, has been validated as a highly potent anti-cancer agent and patented recently as a potential chemotherapeutic drug for various cancers. The aims of this study were, therefore, to investigate the ability of PP in anti-proliferative activity and focused on the lipid profiles revealing the alteration of specific lipid species in the liver fluke *Opisthorchis viverrini* (Ov)-associated cholangiocarcinoma (CCA) cells. PP inhibited CCA cell viability through suppressing mitochondrial membrane potential (MMP) and ATP productions, leading to apoptotic cell death. Liquid chromatography-mass spectrometry combined with chemometrics was performed to investigate lipid alteration during PP-induced apoptosis. The lipidomic analyses showed the altered lipid signatures of CCA cell types including *S*-acetyldihydrolipoamide, methylselenopyruvate, and triglycerides that were increased in PP-treated CCA cells. In contrast, the levels of sphinganine and phosphatidylinositol were lower in the PP-treated group compared with its counterpart. The orthogonal partial-least squares regression analysis revealed that PP-induced MMP dysfunction, leading to remarkably reduced ATP level, was significantly associated with triglyceride (TG) accumulation observed in PP-treated CCA cells. Our findings indicate that PP could suppress the MMP function, which causes inhibition of CCA cell viability through lipid production, resulting in apoptotic induction in CCA cells. These findings provide an anti-cancer mechanism of PP under apoptotic induction ability that may serve as the alternative approach for CCA treatment.

## Introduction

Cholangiocarcinoma (CCA) is a bile duct cancer that originates from the cholangiocyte lining of biliary tracts. CCA is the second most common primary liver cancer worldwide (1). This cancer occurs most commonly in mainland Southeast Asian countries, especially in Northeast Thailand which is strongly associated with the liver fluke *Opisthorchis viverrini* (Ov) infection that has the world's highest incidence rates with the age-standardized incidence varies between 36.3 and 87.7 per 100,000 population in females and males, respectively (2–4). A poor prognosis and low survival rate are burdens for CCA treatment (5, 6). The CCA patients generally undergo surgical resection and chemotherapy, while the effective agents for palliative care are still being explored. Furthermore, CCA has poor response to anti-cancer agents based on characteristics of their multidrug resistant phenotypes, leading to complex mechanisms of chemoresistance (7). Thus, novel therapeutic strategies are necessary for CCA treatment.

Pyrvinium pamoate (PP), a U.S. Food and Drug Administration (FDA)-approved anthelmintic drug, is a NADH-fumarate reductase (FRD) inhibitor in anaerobic organisms such as parasitic helminths or in mammalian cells under a tumor microenvironment, mimicking hypoglycemic and hypoxic conditions. PP reverses the reaction of the mitochondrial electron transport chain complex II, thereby inhibiting ATP production. Under normal aerobic conditions, PP suppresses the mitochondrial electron transport chain complex I and inhibits cancer cell proliferation *via* the JAK/STAT3 signaling pathway (8). Remarkably, many studies have reported that PP is a potent anti-cancer drug against various cancers such as hepatocellular, breast and cervical cancer (9). Moreover, PP was evaluated as a selective Wnt inhibitor that effectively suppressed cell proliferation and metastasis in breast cancer (10, 11).

Lipid metabolism involves many cellular signaling pathways and generates the bioactive lipid molecules that contribute to the regulation of several cellular processes, including cell proliferation, survival, differentiation, apoptosis, inflammation, motility, membrane homeostasis, and chemotherapeutic response (12–15). Alteration of lipids can be used as diagnostic indicators of cancer cell metastasis (16) and the anti-proliferative effects of anti-cancer agents (17). There are bioactive lipid molecules, such as triglyceride, fatty acids, and ceramide, associated with an activation of programmed cell death that can trigger the caspase-dependent apoptotic pathway in many cells and tissues (18–20). However, the diversity of lipids involved is a challenge for qualitative and quantitative lipid analysis. The ultra-high performance liquid chromatography (UHPLC) coupled with electrospray ionization-mass spectrometry (ESI-MS) approach is applicable for lipid profile analysis (21, 22). To date, the cytotoxic activity of PP against CCA cells has not been reported. In the present study, we aimed to determine the inhibitory effects of PP on CCA cell viability. The lipidomic changes of CCA cells in response to PP was elucidated using LC-MS combined with multivariate analysis. The results revealed the types of lipids associated with the cytotoxicity of PP on CCA cells and the regulation of mitochondrial membrane potential (MMP), providing a deeper understanding of the PP mechanism on CCA cell apoptosis.

## Materials and Methods

### Reagents

High-performance liquid chromatography (HPLC)-grade methanol, chloroform and water were purchased from Merck (Darmstadt, DE). Pyrvinium pamoate (PP), dimethyl sulfoxide (DMSO) and sulforhodamine B were purchased from Sigma-Aldrich (St. Louis, MO).

### Cell Culture

The Ov-associated CCA cell lines, KKU-100 and KKU-213A were isolated from the tumor tissues of CCA patients established at the Cholangiocarcinoma Research Institute Khon Kaen University. These cell lines were obtained from the Japanese Collection of Research Bioresources Cell Bank (Osaka, Japan). Primary normal human dermal fibroblast (ATCC® PCS-201-012™) was purchased from American Type Culture Collection (ATCC) (Virginia, US). Cells were cultured in Ham's F-12 nutrient mixture supplemented with 10% heat-inactivated fetal bovine serum (Thermo Fisher Scientific, California, USA), 100 U/mL penicillin and 100 μg/ml streptomycin at 37°C in a humidified incubator containing 5% CO_2_.

### Pyrvinium Pamoate Cytotoxicity

Cells were plated into 96-well flat-bottom microtiter plates at 2 × 10^3^ cells/mL and allowed to adhere for 12 h. PP was prepared in DMSO. Cells were incubated in PP at different concentrations (20, 40, 80, 160, 320, 640, 1,280, and 2,560 nM) for 48 h. Untreated cells were incubated in culture media with 0.1% DMSO. Doses of PP were selected based on half maximal inhibitory concentration (IC50). Cell viability was determined by sulforhodamine B (SRB) assay.

### Cell Viability Assay

An SRB assay was used for determining the viability of cells. The cells were fixed with 10% (v/v) trichloroacetic acid followed by the addition of 0.4% (w/v) SRB in 1% (v/v) acetic acid. The protein-bound stain was solubilized with 10 mM Tris base at pH 10.5. Absorbance was measured at 540 nm using a microplate reader (TECAN Trading, Männedorf, CH).

### Apoptotic Cell Staining

KKU-100 and KKU-213A cells were plated into cell culture chamber slides at 4 × 10^4^ cells/mL. The cells were treated with PP (80, 160, and 320 nM) for 12, 24, 36, and 48 h. Apoptotic cells were stained using an annexin-V and propidium iodide staining kit according to the manufacturer's instruction (Roche, Basel, CH). The nuclei were labeled with Hoechst 33342 (Invitrogen, California, US). Stained cells were visualized by confocal laser scanning microscopy (Zeiss LSM 800, Carl Zeiss, DE).

### Western Blot Analysis

KKU-100 and KKU-213A cells were plated into 6-well flat-bottom plates at 5 × 10^4^ cells/mL. The cells were treated with PP for 48 h. Cell lysates were extracted with RIPA lysis buffer (150 mM NaCl, 0.5 M Tris-HCl pH 7.4, 1% (v/v) Tween-20, 1% (w/v) sodium deoxycholate, 0.1% (w/v) SDS). Protein concentration was determined using the Pierce™ BCA Protein Assay Kit (Thermo Fisher Scientific, California, USA). Protein extracts containing 20 μg protein was solubilized in 4x SDS buffer containing β-mercaptoethanol and boiled at 95°C. Protein were electrophoresed on 10% polyacrylamide gel by SDS-PAGE then transferred to polyvinylidene difluoride membranes. The membranes were incubated in 5% skim milk for 1 h at room temperature and then probed at 4°C overnight with the following antibodies: rabbit anti-human Bcl-2 (Cell Signaling Technology, Massachusetts, US), mouse anti-human Bax (BD Biosciences, San Jose, CA) and mouse-anti-human β-actin (Invitrogen, California, US). β-actin was used as a loading control. After incubation with the respective secondary antibody (Abcam, Cambridge, UK), the band intensity was detected by ECL™ Prime Western Blotting Detection Reagent (GE Healthcare, Illinois, USA) for chemiluminescent detection. The apparent density of the bands on membranes was captured by ImageQuant™ Imager (GE Healthcare, Illinois, US).

### Mitochondrial Membrane Potential Assay

KKU-100 and KKU-213A cells were plated into 24-well flat-bottom plates at 2 × 10^4^ cells/mL. The cells were treated with PP for 48 h. Cells were stained using a TMRE-MMP Assay Kit according to the manufacturer's instructions (ab113852, Cambridge, UK). Then, the cells were visualized by confocal microscopy (Zeiss LSM 800, Carl Zeiss, DE).

### ATP Measurement

The cells were plated into 96-well flat-bottom black plates at 2 × 10^4^ cells/mL. After incubation with PP for 48 h, ATP level in the treated cells was measured using the CellTiter-Glo® Luminescent Cell Viability Assay (Promega, Wisconsin, US). ATP disodium salt hydrate (Sigma-Aldrich, St. Louis, MO) was used as a standard. The luminescence was measured at 470 nm using SpectraMax® microplate readers (MDS Analytical Technologies, USA).

### Assessment of Lipid Droplet Content

CCA cells were plated into cell culture chamber slide at 4 × 10^4^ cells/mL and treated with PP for 12, 24, 36, and 48 h. Cells were stained with 2 μM of BODIPY™ 493/503 (4,4-Difluoro-1,3,5,7,8-Pentamethyl-4-Bora-3a,4a-Diaza-s-Indacene) (Invitrogen, California, US) at 37°C for 15 min. Cells were washed twice with PBS and viewed under confocal microscopy.

### Sample Collection and Preparation for LC-MS Lipid Profiling

KKU-100 and KKU-213A were seeded at 2 × 10^5^ cells/mL into 100-mm cell culture dishes. After treatment with PP for 48 h, the treated cells (2 × 10^6^ cells of each condition) were collected by detaching them from dish using trypsin-EDTA and then centrifuged at 2,000 rpm at 4°C for 5 min. The cell pellets were washed with PBS 3 times to remove the remaining medium and immediately frozen with liquid nitrogen (LN_2_). For lipid extraction, the frozen cells were resuspended in methanol and sonicated at 40% amplitude for 3 cycles. The lysed cells were phase extracted using water/methanol/chloroform (1:1:3 v/v), incubated on ice for 20 min and centrifuged at 4,000 rpm at 4°C for 20 min. The organic phase was collected and transferred to a glass vial tube (KIMA, Arzegrande, IT), followed by drying the solvent under a fume hood overnight. The dried lipid extracts were reconstituted in a 200 μL solvent mixture of isopropanol (IPA)/acetonitrile (ACN)/H_2_O (2:1:1 v/v), and centrifuged for 20 min at 13,000 × g, 4°C. For each sample, 20 μL was collected, pooled, and then used as a quality control (QC) sample for each interval throughout the analysis.

### LC-MS Data Acquisition

Lipid analysis was carried out by the Khon Kaen University International Phenome Laboratory (KKUIPL) using ultra-high performance liquid chromatography coupled with electrospray ionization-quadrupole time-of-flight mass spectrometry (compact UHPLC ESI-Q-TOF MS, Bruker, DE). Briefly, the samples were injected into the UHPLC system equipped with a Bruker intensity C18 column (100 × 2.1 mm, 1.9 μm). The column temperature was set at 40°C. Mobile phase A was composed of acetonitrile (ACN)/water (60:40 v/v), and mobile phase B, isopropanol (IPA)/ACN (90:10 v/v) with 10 mM ammonium formate and 0.1% w/v formic acid in both. The elution gradient was set as follows: 40% B (0–2 min), 43% B (2–2.1 min), 50% B (2.1–12 min), 70% B (12.1–18 min), 90% B (18–18.1 min) and 40% B (18.1–20 min) with a flow rate of 0.4 mL/min. A pooled sample (QC) was injected 10 times before initiating the batch run to condition the column. Then, the QC sample was reinjected at the beginning, every five sample injections, and at the end to estimate the instrument stability and reproducibility. Two μL of samples were used for both positive and negative ionization modes. The MS system was set at 220°C, desolvation flow gas at 8 L/min and time of flight mass spectrometry (TOF) with the Apollo II electrospray ionization (ESI) as the ion source. The ionization voltage in positive and negative polarity modes were 4,000 and 4,500 V, respectively. Data were acquired in the profile mode for 20 min, and the scan range was set at 50–1,300 m/z. Tandem mass spectrometry (MS/MS) was performed to QC with the sample pooled to identify lipid species.

### Data Processing

All spectra were processed using Metaboscape 5.0 software (Bruker, Massachusetts, US). The raw data files (.d) were converted to.CSV files including a list of retention times, peak intensities and m/z values. The data-dependent MS/MS was employed on Metaboscape (Bruker, Germany) and MS-DIAL software version 4.20 (RIKEN Center for Sustainable Resource Science, Kanagawa, JP) for assigning lipid species through fragmentation patterns. Public databases including Metlin (https://metlin.scripps.edu), Human Metabolome Database (HMDB) (https://hmdb.ca) and LipidMAPS (https://www.lipidmaps.org) were also used. The level of identification was determined using the outline in Sumner et al. (23).

### Statistical Analysis

The coefficient of variation (CV) based on the QC samples was calculated for filtering the different variables. Variables with CV >30% were removed from the dataset. Principle component analysis (PCA), orthogonal partial least squares (O-PLS) and O-PLS for discriminant analysis (O-PLS-DA) were used for data modeling and statistical analysis on SIMCA software version 14.1 (Umetrics, Umeå, SE) and data was scaled using Pareto scaling. S-plot analysis was employed to determine the discriminatory metabolites between groups. CV-ANOVA was used to evaluate significant differences. The lipid intensities were calculated and used to determine significant differences through unpaired *t*-test (*p* < 0.05) on GraphPad Prism 8 software (California, US).

## Results

### PP Inhibited Viability of CCA Cells

**Figure 1:**
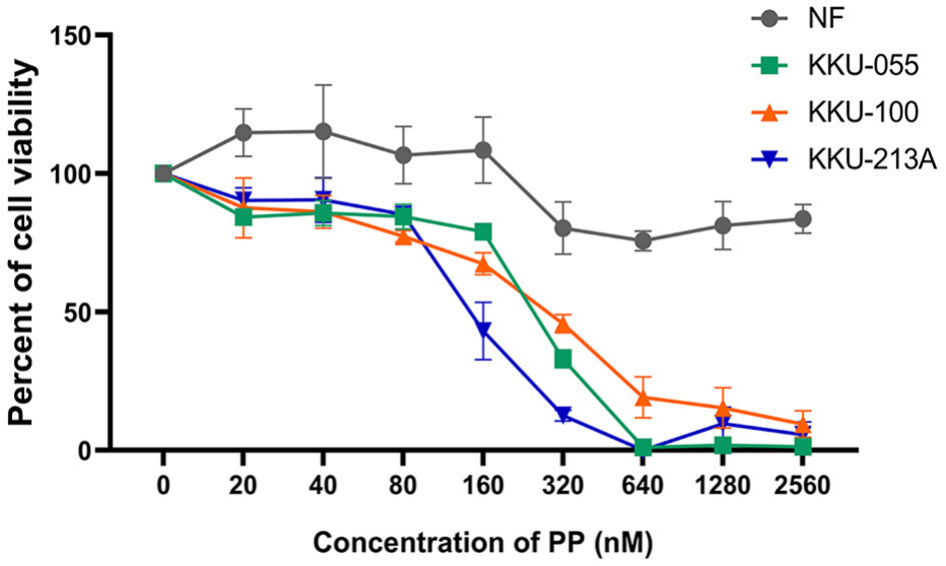
The inhibitory effect of PP on CCA cell viability. KKU-055, KKU-100, KKU-213A cells and primary normal human dermal fibroblasts (NF) were treated with various concentrations of PP for 48 h. DMSO 0.1% (v/v) was used as a control. Cell viability was determined by SRB assay. Error bars represent the standard deviation (SD) of triplicate experiments. PP, pyrvinium pamoate.

The cytotoxic effect of PP on CCA cell lines (KKU-055, KKU-100 and KKU-213A) and a primary normal human dermal fibroblast (NF) was determined using a SRB assay, at various PP concentrations (80–320 nM) for 48 h. We found that PP decreased CCA cell viability in a dose-dependent manner, whereas PP had no effect on NF (Figure 1). The half-maximal inhibitory concentrations (IC50) of PP on KKU-055, KKU-100 and KKU-213A cells were 245.9 ± 9.4, 279.7 ± 28.5, and 148.7 ± 27.2 nM, correspondingly. According to their IC50, we selected KKU-100 (less sensitive to PP) and KKU-213A (highly sensitive to PP) for further investigation.

### PP Induced Apoptosis in CCA Cells

**Figure 2:**
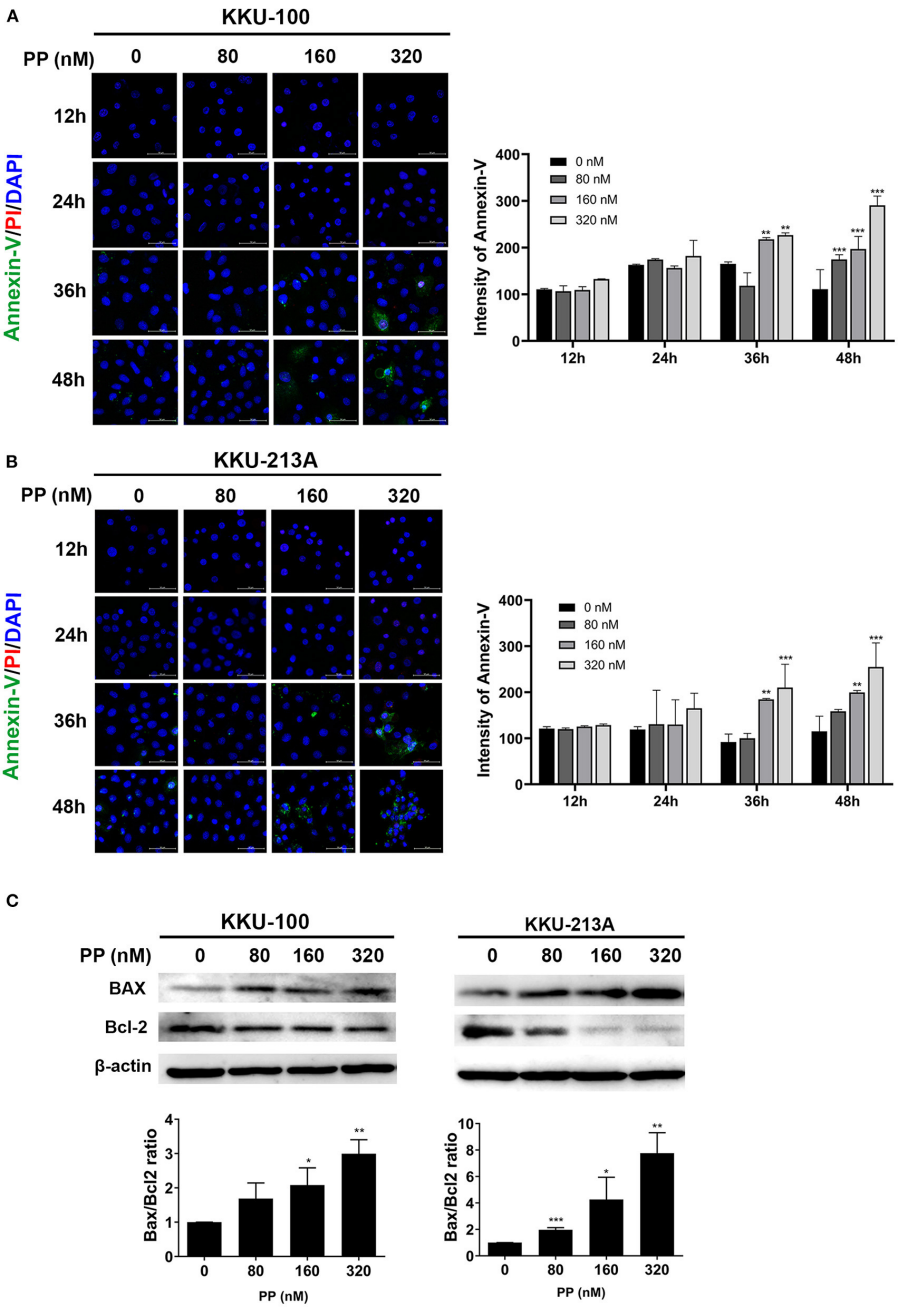
The effect of PP on apoptotic cell death in the CCA cell lines. KKU-100 and KKU-213A were treated with PP for 48 h. **(A,B)** CCA apoptotic cells were demonstrated by annexin V-FITC and propidium iodide double staining using confocal microscopy. After treatment, the cells were incubated with annexin V-FITC and propidium iodide following the manufacturer's protocol. Hoechst 33342 was used to visualize the nuclei of CCA cells. Green, stained with annexin V-FITC; red, stained with propidium iodide and blue, stained with Hoechst 33342. Original magnification is 400x. **(C)** Western blot analysis shows effect of PP on CCA cells apoptosis by increasing the BAX/Bcl-2 ratio. Bax and Bcl-2 levels were assessed after PP treatment for 48 h from KKU-100 and KKU-213A cell lysates. Error bars represent the standard deviation (SD) of triplicate experiments. The significant difference was determined using unpaired *t*-tests (*p < 0.05, **p < 0.01, ***p < 0.001) compared to the control group.

To demonstrate the multitude of morphological and biochemical features of apoptotic cells, we performed annexin-V and propidium iodide staining to identify the apoptotic cells. After 48-h treatment with PP, KKU-100 and KKU-213A cells were incubated with annexin-V and propidium iodide and the stained cells were observed by confocal microscopy. The result showed that the untreated cells did not display annexin-V and propidium iodide staining, while the PP-treated cells showed increasing annexin-V (green) and propidium iodide (red) staining from 80 to 320 nM in Figures 2A,B. The annexin-V staining was slightly increased at 36 h in PP-treated cells at 160 nM and markedly increased at 48 h in PP-treated cells at 320 nM. The nuclei of CCA cells were stained with Hoechst 33342 as shown in blue. Additionally, the BAX/Bcl-2 ratio, a measurement that indicates apoptotic induction, was assessed using western blotting. The result showed that treated cells had a significantly increased BAX/Bcl-2 ratio when compared with the control group (Figure 2C). These results indicate that PP induced apoptotic cell death in CCA cells.

### Lipidomic Profiles of PP-Treated CCA Cells Display Distinctive Changes

**Figure 3:**
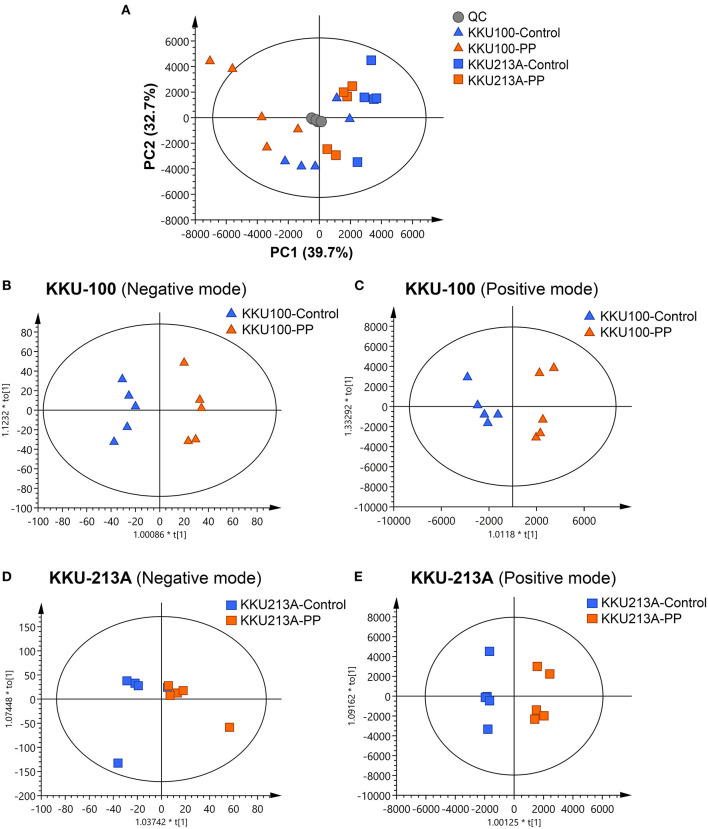
Multivariate analysis of the lipidomic profiles of PP-treated CCA cells. **(A)** The PCA score plot of positive and negative modes of PP-treated and control groups in KKU-100 and KKU-213A cells (*n* = 5 for each group). The O-PLS-RE score plot based on cytotoxicity of **(B,C)** KKU-100 and **(D,E)** KKU-213A cells in control vs. PP-treated group models.

In this study, lipid profiling of PP-induced apoptosis in CCA cells for 48 h was investigated using LC-MS/MS analysis. Spectral data obtained from non-polar extracts of PP-treated and control groups were compared using multivariate statistical analysis. Principle component analysis (PCA) was employed to distinguish metabolite contents between the control and PP treated group of each CCA cell line in positive and negative ionization modes. Quality control (QC) samples were included to assess the function of the apparatus. The PCA score plot showed that the KKU-100 cells were likely separated from the KKU-213A cells, with 39.7% of the variance in data explained by principal component 1 (PC1), 32.7% by PC2 and 12.1% by PC3, and Q2 = 0.710 (Figure 3A, Supplementary Figure 1).

**Figure 4:**
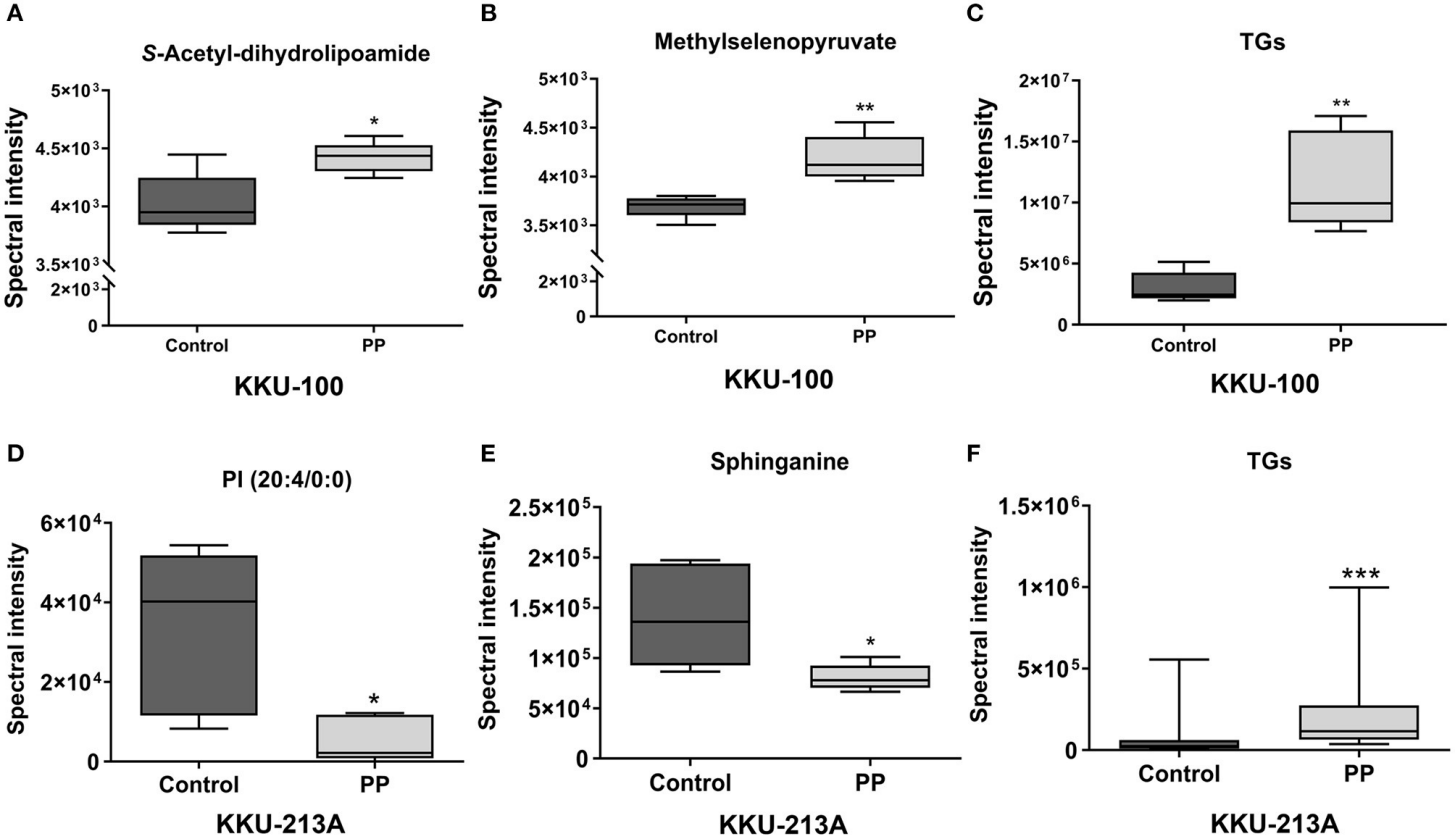
Relative concentrations of candidate lipids. **(A)** *S*-acetyl-dihydrolipoamide, **(B)** methylselenopyruvate, **(C)** TG species (TGs) of KKU-100 cells after treatment with PP. Candidate lipids of PP-treated KKU-213A cells including **(D)** PI (20:4/0:0) **(E)** sphinganine and **(F)** TG species. Error bars represent the standard deviation (SD) of samples (*n* = 5). The significant difference was determined using unpaired *t*-tests (*p < 0.05, **p < 0.01, and ***p < 0.001) compared to control group.

Pairwise comparisons were analyzed using the orthogonal partial-least squares for regression analysis (O-PLS-RE) based on the cytotoxicity of the control vs. the PP-treated groups in each positive and negative mode for both KKU-100 and KKU-213A cells (Figures 3B–E). We found that the negative and positive mode of KKU-100 cells and positive mode of KKU-213A cells represented the models with the highest goodness of fit and predictability, as indicated by the parameters R^2^X and Q^2^Y as shown in Supplementary Figure 1. The model validity was determined using 100 times permutation test (*p* < 0.05). Next, an S-plot was calculated to determine the lipids that contribute to class separation between the groups. In this study, the candidate variables that represented the lipids' response to PP-induced apoptotic cell death were selected based on a principle VIP value of more than 1.0 and p (corr) cutoff value of 0.6. The results showed that upon treatment with PP, KKU-100 cells had increased levels of S-acetyldihydrolipoamide, methylselenopyruvate and triglyceride species (TGs) (Figures 4A–C). Meanwhile, KKU-213A cells had a significant decreased level in phosphatidylinositol (PI) (20:4/0:0) and sphinganine levels but had a markedly increased level in TGs (Figures 4D–F). The identified lipid species and the fragmentation mass spectra are listed in Supplementary Table 1.

### PP Suppresses Mitochondrial Membrane Potential and ATP Levels of CCA Cells

**Figure 5:**
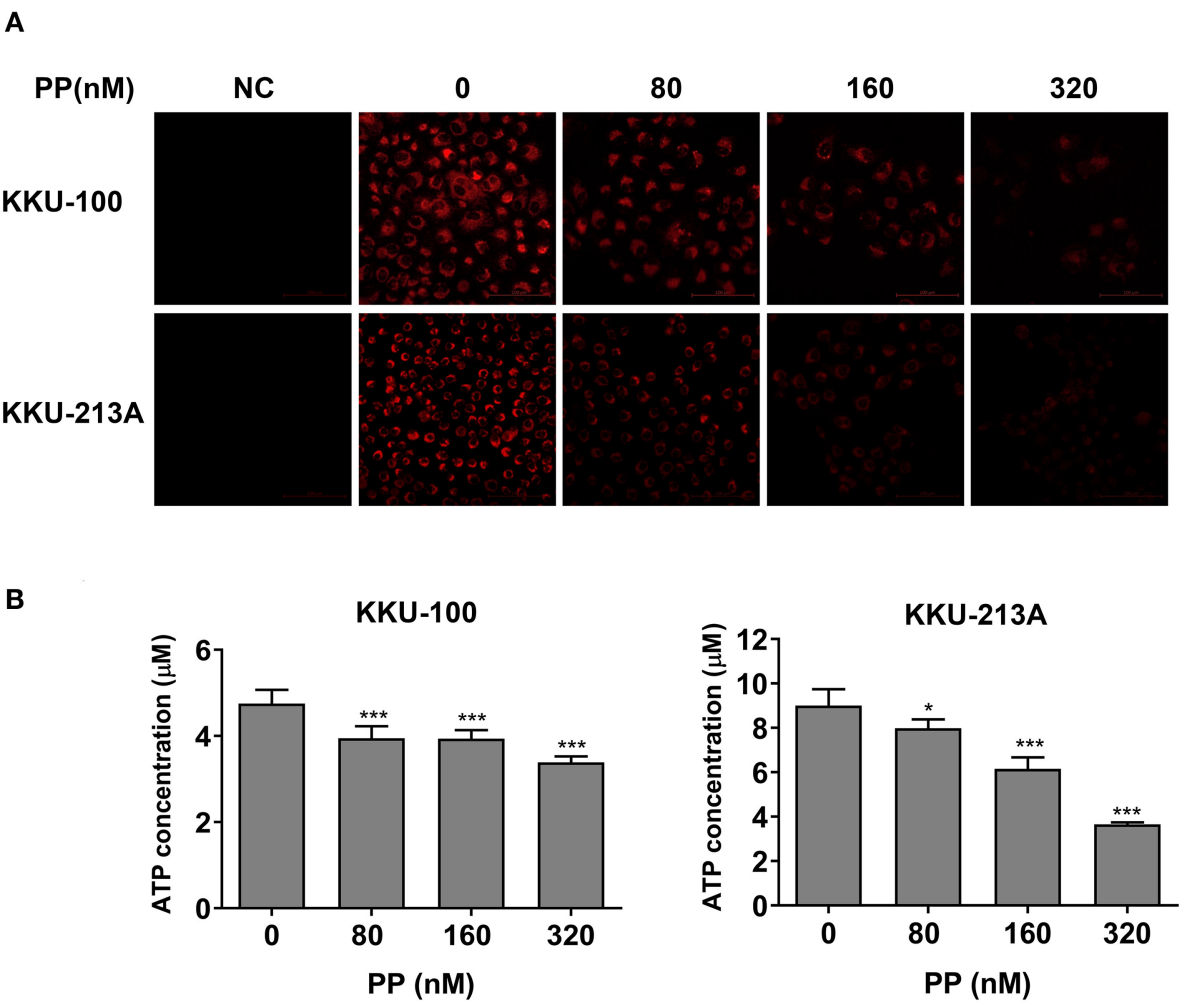
The inhibitory effect of PP on mitochondrial membrane potential and ATP level of CCA cells. KKU-100 and KKU-213A cells were treated with PP for 48 h. **(A)** The cells were incubated with TMRE and then viewed under confocal microscopy. Negative control cells were incubated with FCCP before TMRE staining. **(B)** ATP levels of KKU-100 and KKU-213A were measured using the CellTiter-Glo® luminescent cell viability assay. The ATP concentrations at 0.01, 0.1, and 1 μM were used to create a standard curve. Error bars represent the standard deviation (SD). The significant difference was determined using unpaired *t*-tests (*p < 0.05, ***p < 0.001) compared to control group. NC, negative control.

To determine the effect of PP on the apoptosis of CCA cells, we hypothesized that PP could suppress the cells' mitochondrial function, resulting in lipid accumulation in apoptotic cells. Therefore, we investigated the mitochondrial membrane potential (MMP) of CCA cells upon PP treatment for 48 h using the tetramethylrhodamine ethyl ester (TMRE)-MMP assay of which TMRE is a specific indicator of membrane potential. Carbonyl cyanide-4-(trifluoromethoxy) phenylhydrazone (FCCP) was used as a negative control. We found that TMRE was highly accumulated in untreated cells as shown in red, while its intensity was dose-dependently decreased in the treated group (Figure 5A). TMRE-positive staining was not observed in the negative control group. Additionally, the cellular ATP content of CCA cells was measured after treatment with PP using the CellTiter-Glo® luminescent cell viability assay. The result showed that PP significantly suppressed the ATP content in both CCA cell lines (Figure 5B), indicating that PP inhibited the MMP in CCA cells.

### O-PLS Regression Analysis Reveals the Correlation of PP-Suppressed Mitochondrial Membrane Potential and TG Accumulation in CCA Cells

**Figure 6:**
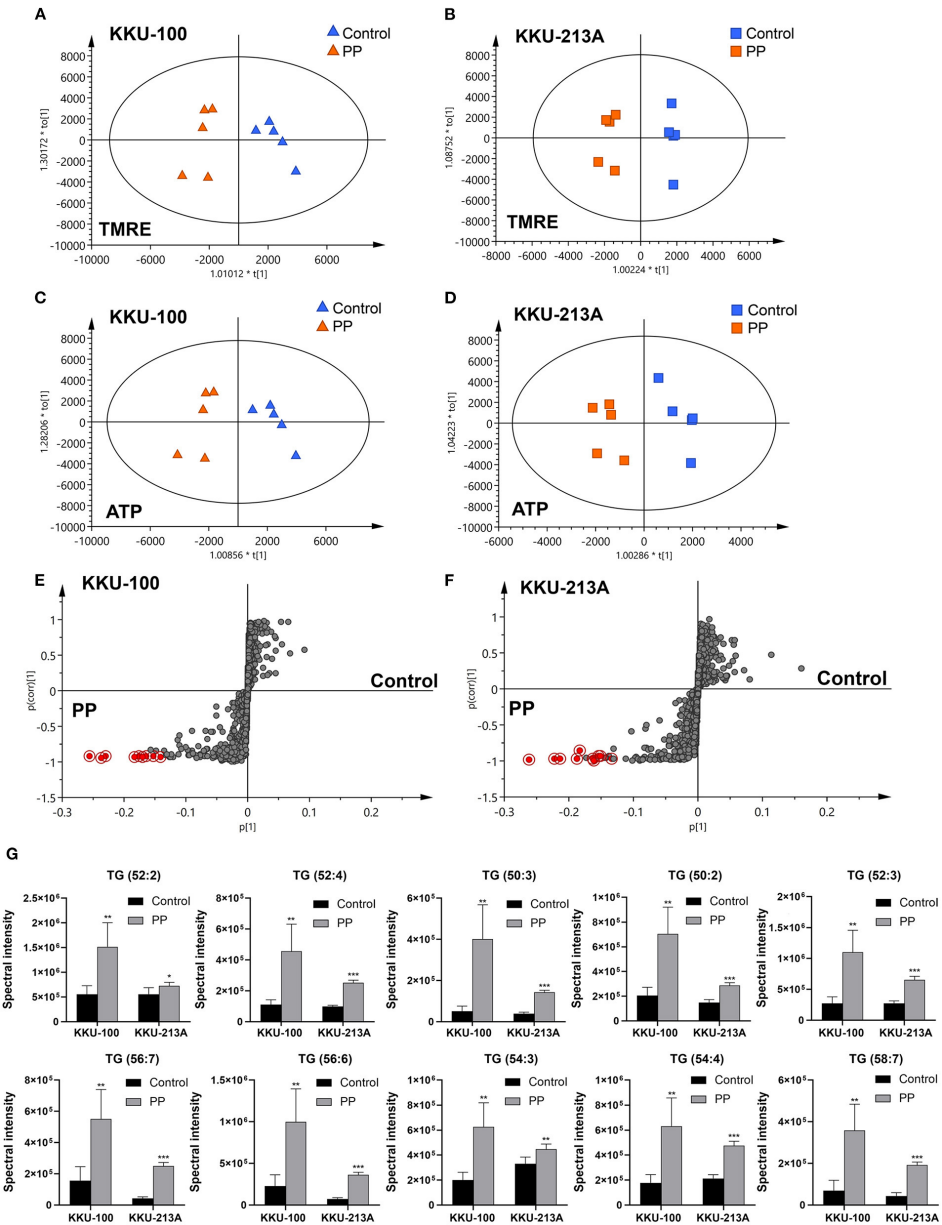
O-PLS regression analysis of the lipidomic profiles with mitochondrial membrane potential in PP-treated CCA cells. O-PLS regression score plot based on TMRE intensities and ATP levels in **(A,C)** KKU-100 and **(B,D)** KKU-213A cells, respectively. The S-plots derived from the altered lipid profiling of **(E)** KKU-100 and **(F)** KKU-213A cells in control vs. PP treatment groups. **(G)** Relative concentrations of candidate lipids associated with ATP levels and TMRE intensities in KKU-100 and KKU213 cells. Error bars represent the standard deviation (SD) of samples (*n* = 5). The significant difference was determined using unpaired *t*-tests (*p < 0.05, **p < 0.01, ***p < 0.001) compared to control group.

O-PLS regression analysis was performed using the lipidome and MMP data obtained from the PP-treated and control groups. The O-PLS regression analysis of lipidome against the raw intensities of TMRE and ATP content levels showed that PP-treated groups were significantly separated from the control group in both KKU-100 and KKU-213A CCA cells (Figures 6A–D). The S-plots derived from the altered lipid profiles were determined based on the VIP values. The selected candidate variables are colored in red, representing the lipids with increased levels after PP treatment in association with the inhibition of MMP and ATP content (Figures 6E,F). We found that levels of TGs: TG (52:2), TG (52:4), TG (50:3), TG (50:2), TG (52:3), TG (56:7), TG (56:6), TG (54:3), TG (54:4), and TG (58:7) were significantly increased in the PP-treated CCA cells when compared to the control group (Figure 6G). This suggests that the suppression of MMP and ATP content by PP could lead to the accumulation of TGs in CCA cells. The candidate lipid species and fragmentation mass spectra are listed in Supplementary Table 2.

### PP Increases Lipid Droplet Accumulation in CCA Cells

**Figure 7:**
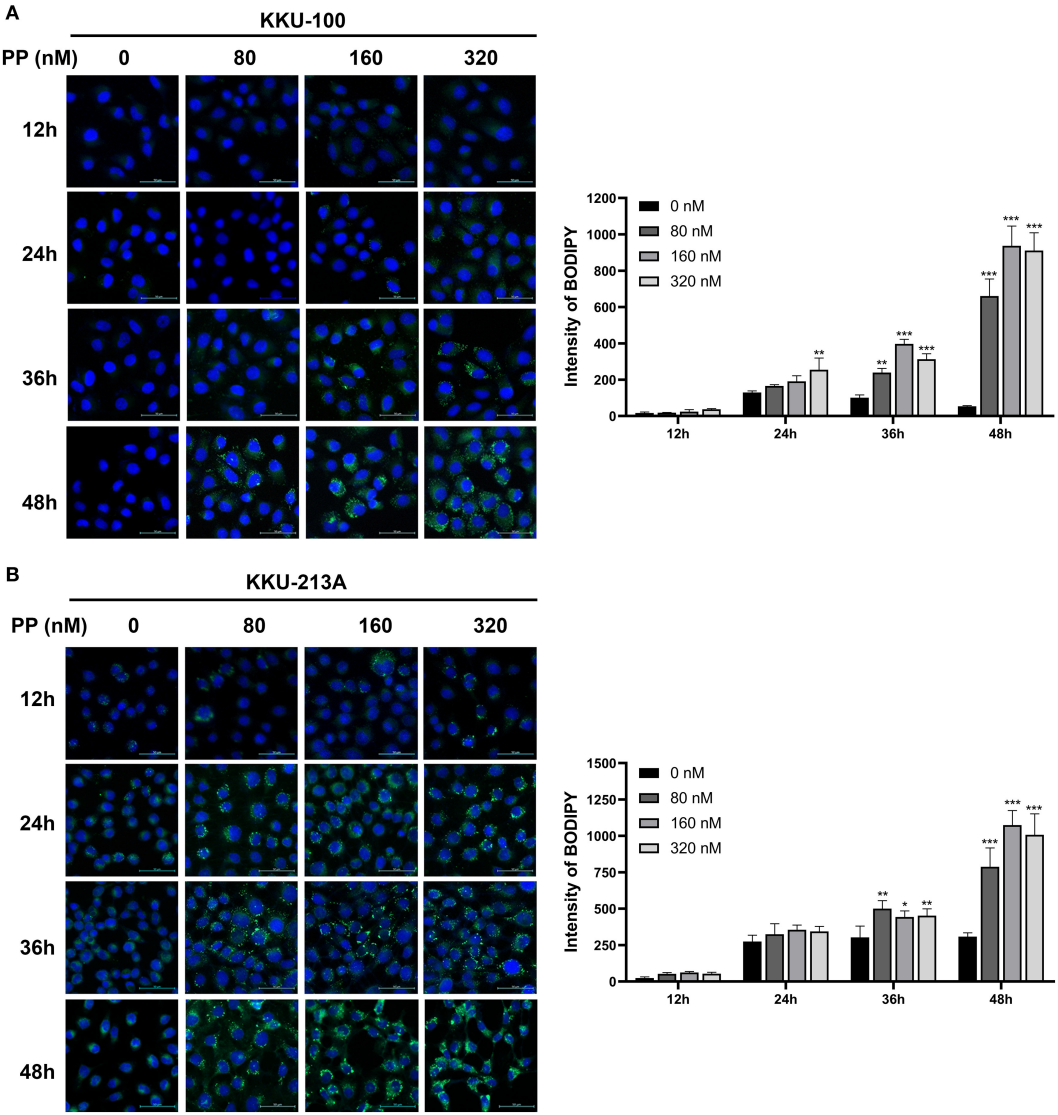
Lipid droplet staining in CCA cells. **(A)** KKU-100 and **(B)** KKU-213A cells were treated with PP for 12, 24, 36, and 48 h. The cells were incubated with BODIPY™ 493/503 to stain neutral lipid. The stained cells were observed under confocal microscopy. Intensities of lipid droplet in KKU-100 and KKU-213A cells. Error bars represent the standard deviation (SD). The significant difference was determined using unpaired *t*-tests (*p < 0.05, **p < 0.01, ***p < 0.001) compared to control group.

To investigate the effect of PP on TG accumulation of CCA cells, we performed BODIPY 493/503 staining to stain neutral lipid formation such as TGs in CCA cells. Lipid droplets were observed as shown in green dots (Figures 7A,B). The staining intensities of TGs tended to be increased in PP-treated CCA cells at 24-h post incubation and significantly increased at 36 and 48 h in a dose-dependent manner when compared to control group. Our findings reveal that PP-induced apoptosis co-committed with increased lipid droplet accumulation in CCA cells that are consistent with our LC-MS lipidomic results.

## Discussion

PP has been reported as a potent anti-cancer drug in various cancers (9). It suppresses mitochondrial electron transport chain complex activities, affects several signaling pathways such as JAK/STAT3 and Wnt, and inhibits cancer cell proliferation (8). In this study, we demonstrated the mechanism of PP in the suppression of CCA cell growth *in vitro*. We found that PP is an effective anti-proliferative agent by inhibiting CCA cell viability and inducing apoptosis. In addition, lipid alterations in CCA cells in response to PP-induced apoptosis were determined by performing untargeted lipidomic analysis using the UHPLC-MS/MS technique combined with multivariate statistical analysis.

Lipidomic analysis revealed that the major lipid alterations induced by PP treatment are associated with apoptotic signaling. The accumulation of lipid species in KKU-100 cells includes *S*-acetyldihydrolipoamide and methylselenopyruvate that are increased after treatment with PP. Previous studies showed that S-acetyldihydrolipoamide is found in gastric cancer cells and is associated with trastuzumab response (24). Methylselenopyruvate is an α-keto acid metabolite of methylselenocysteine which acts as a histone deacetylase 8 (HDAC8) inhibitor that can restore Bcl2 modifying factor (BMF) downregulation and thereby activates apoptosis in colon cancer cells (25). In addition, sphinganine, which was decreased in PP-treated KKU-213A cells, can be acylated to generate ceramide, which is involved in cellular apoptotic responses (26). PP-treated KKU-213A cells decreased in PI level. PI can be phosphorylated by lipid kinase, leading to the production of phosphoinositides that are involved in many signaling pathways mediating cell proliferation and survival, such as the PI3K/AKT pathway (27). Activation of the PI3K/AKT signaling pathway plays an important role in CCA progression. PI3K/mTOR inhibitors can suppress CCA cell growth and present a possible therapeutic target for CCA treatment (28). This result implies that PP may serve as the potential agent for suppressing the PI3K/AKT signaling pathway in CCA cells. Furthermore, the altered lipids found in both PP-treated KKU-100 and KKU-213A were triglyceride species (TGs). We proved that the onset of TG accumulation in PP-treated cells occurred at the early apoptosis (24-h treatment). The accumulation of TGs was described as the apoptotic features and response to an activation of apoptosis (17, 29). The macrophages lacking adipose triglyceride lipase (ATGL), which caused spontaneous induction of apoptosis due to energy deprivation because of lipolysis defection, showed increasing of intracellular TG concentration compared with wild-type macrophages. Incubation of VLDL in wild type macrophages to achieve similar TG loading in apoptotic cells, VLDL-loaded macrophages were increased number of apoptotic cells and apoptotic protein markers (30). In addition, TG treatment in THP-1 monocyte inhibited cell proliferation mediated by inducing production of TNF-α, a cytokine involved in inflammation and promotion of apoptosis, to enhance cell death (31). Excess free fatty acids (FFAs) cause endoplasmic reticulum (ER) stress through accumulation of saturated TG leading to severe disruption of ER architecture that would contribute to cell death (32). These studies suggested that TG accumulation might trigger apoptotic death in CCA cells.

Besides, our findings show that PP can suppress MMP and ATP contents in CCA cells. The mechanism of PP as a suppressor of the mitochondrial electron transport chain complexes I and II, leads to the inhibition of ATP production which in turn leads to cancer cell death as previously reported (8, 9). The inhibitory effect of PP given on mitochondrial function could alter source of lipid metabolism and energy supply in CCA cells. The O-PLS regression analysis showed that the reduction of TMRE intensities and ATP levels was associated with TG production in the PP-treated group for both CCA cell lines. Validation of the elevated neutral lipid formation such as TGs, were also clearly shown in PP-treated CCA cells. The TG formation was observed in time-dependent exposure of PP treatment suggesting that TGs involve in the mechanism of apoptosis in CCA cells. Inhibition of mitochondrial fatty acid oxidation caused apoptosis in murine lymphoma cells, increased TG synthesis by incorporation of fatty acids into TGs leading to increasing of lipid droplet formation in cytoplasm during the apoptotic response which occurs as downstream of p53 activation and inhibition of mTOR signaling pathway (33). The macrophage lacking ATGL with cellular TG accumulation showed decreasing of total acyl Co-A and acyl-carnitine concentrations leading to impaired energy metabolism (34) and defective of lipolysis causes intracellular lipid droplets resulting in mitochondrial fragmentation, loss of membrane potential, reduced oxygen consumption and elevated cytosolic Ca^2+^ levels and ROS production contributing to activation of the mitochondrial apoptosis pathway (30). These studies suggest that mitochondrial dysfunction could activate apoptotic cell death by increasing intracellular TG accumulation. Therefore, we conclude that PP can suppress mitochondrial function causing the inhibition of CCA cell viability through lipid accumulation leading to apoptotic cell death.

**Figure 8:**
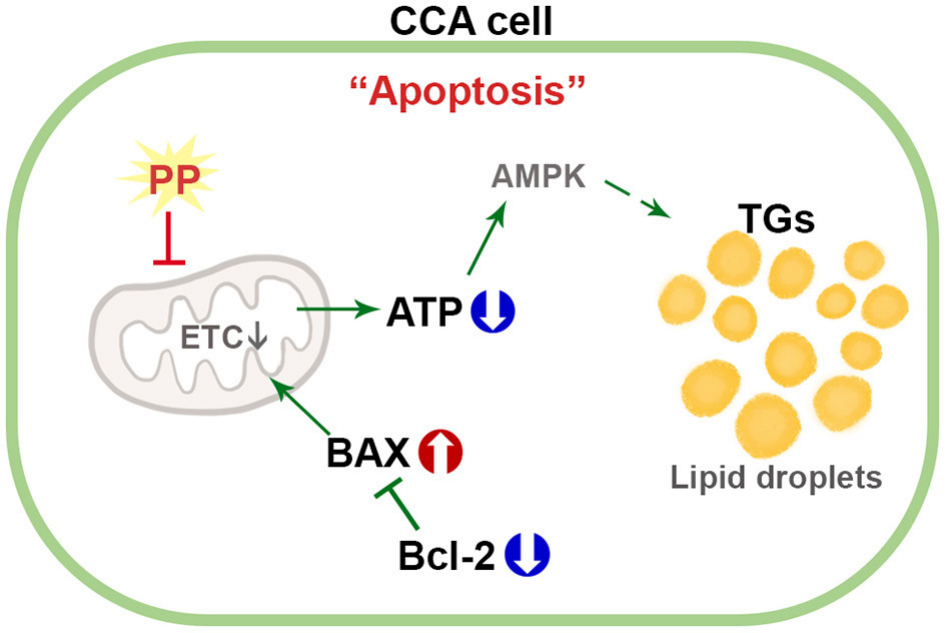
Schematic diagram for the mechanism of PP in apoptotic induction by increasing lipid accumulation and inhibiting mitochondrial function in CCA cells.

This study distinctly demonstrates the mechanism of action of PP in the induction of apoptotic death in Ov-associated CCA cells. Our results show that PP treatment suppresses CCA cell viability by inhibiting mitochondrial function leading to ATP depletion. This event affects lipid accumulation and apoptosis induction. To our knowledge, this is the first study to evaluate the anti-cancer potential of PP on CCA cells and demonstrate the lipid profiles in PP-treated CCA cell lines (Figure 8). *In vivo* studies, however, need to be conducted to assess the toxicity and mechanisms underlying the anti-proliferative activity and apoptotic response to further help elucidate its anti-cancer potential. Collectively, the findings obtained from the current study shed light on the potential of PP toward alternative strategies for CCA treatment.

## Data Availability Statement

The original contributions presented in the study are included in the article/Supplementary Material, further inquiries can be directed to the corresponding author/s.

## Author Contributions

NN provided the concept for the research. YK and ST designed the study. YK performed the main experiments. YK, JP, and BP analyzed the manuscript data. YK and NN wrote the paper. All authors discussed the data.

## Funding

This work was supported by the Thailand Research Fund through Khon Kaen University and The Royal Golden Jubilee Ph.D. Program (Grant no. PHD/0215/2560) to YK and NN, a grant from Faculty of Medicine, Khon Kaen University (Grant no. IN62305), and the NSRF under the Basic Research Fund of Khon Kaen University under through Cholangiocarcinoma Research Institute to NN.

## Conflict of Interest

The authors declare that the research was conducted in the absence of any commercial or financial relationships that could be construed as a potential conflict of interest.

## Publisher's Note

All claims expressed in this article are solely those of the authors and do not necessarily represent those of their affiliated organizations, or those of the publisher, the editors and the reviewers. Any product that may be evaluated in this article, or claim that may be made by its manufacturer, is not guaranteed or endorsed by the publisher.

## Supplementary Material

Supplementary Figure 1 Multivariate analysis of the lipidomic profile of PP-treated CCA cells in negative and positive modes, related to Figure 3. **(A,C)** PCA (R^2^X = 0.883, Q^2^ = 0.0843, and R^2^X = 0.904, Q^2^ = 0.788, respectively) **(B,D)** O-PLS-RE score plot (R^2^X = 0.757, Q^2^Y = 0.885, and R^2^X = 0.727, Q^2^Y = 0.693, respectively) of KKU-100 with PP-treated and control groups in negative and positive modes. **(E,G)** PCA (R^2^X = 0.862, Q^2^ = 0.177, and R^2^X = 0.899, Q^2^ = 0.792, respectively) and **(F,H)** O-PLS-RE score plot (R^2^X = 0.836, Q^2^Y = 0.438, and R^2^X = 0.801, Q^2^Y = 0.901, respectively) of KKU-213A with PP-treated and control groups in negative and positive modes, respectively (*n* = 5 for each group).

Supplementary Table 1 The altered lipid species identification for KKU-100 and KKU-213A cells after treatment with PP in positive and negative modes, related to Figure 3.

Supplementary Table 2 The candidate lipid species identification of KKU-100 and KKU-213 cells associated with mitochondrial membrane potential after treatment with PP, related to Figure 6.
