# Supplementary figures and images for: Lipidomic Analyses Uncover Apoptotic and Inhibitory Effects of Pyrvinium Pamoate on Cholangiocarcinoma Cells via Mitochondrial Membrane Potential Dysfunction
Source: Front Public Health. 2021 Dec 7;9:766455. doi: 10.3389/fpubh.2021.766455 (PMC8688698; doi:10.3389/fpubh.2021.766455)

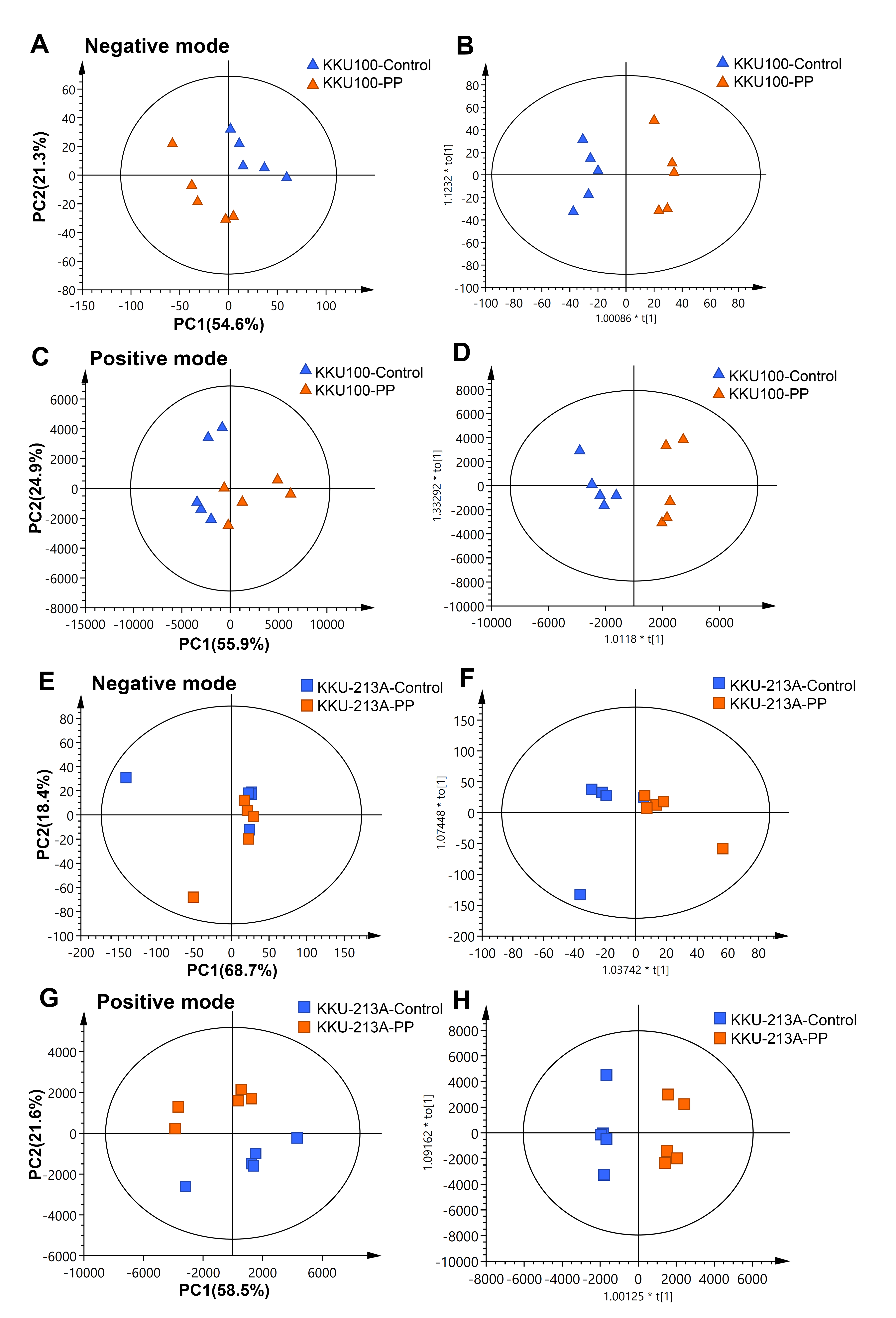

Supplement: Supplementary Figure 1 — Multivariate analysis of the lipidomic profile of PP-treated CCA cells in negative and positive modes, related to Figure 3. (A,C) PCA (R2X = 0.883, Q2 = 0.0843, and R2X = 0.904, Q2 = 0.788, respectively) (B,D) O-PLS-RE score plot (R2X = 0.757, Q2Y = 0.885, and R2X = 0.727, Q2Y = 0.693, respectively) of KKU-100 with PP-treated and control groups in negative and positive modes. (E,G) PCA (R2X = 0.862, Q2 = 0.177, and R2X = 0.899, Q2 = 0.792, respectively) and (F,H) O-PLS-RE score plot (R2X = 0.836, Q2Y = 0.438, and R2X = 0.801, Q2Y = 0.901, respectively) of KKU-213A with PP-treated and control groups in negative and positive modes, respectively (n = 5 for each group). [file Image_1.TIF]
