# Supplementary material for: Lipidomic Analyses Uncover Apoptotic and Inhibitory Effects of Pyrvinium Pamoate on Cholangiocarcinoma Cells via Mitochondrial Membrane Potential Dysfunction
Source: Front Public Health. 2021 Dec 7;9:766455. doi: 10.3389/fpubh.2021.766455 (PMC8688698; doi:10.3389/fpubh.2021.766455)
Supplement: Supplementary Table 1 — The altered lipid species identification for KKU-100 and KKU-213A cells after treatment with PP in positive and negative modes, related to Figure 3. [file Table_1.DOCX]

Supplementary Material

**Table S1.** The altered lipid species identification for KKU-100 and KKU-213A cells after treatment with PP in positive and negative modes, related to Figure 3.

| No. | Lipid molecular species | No. | m/z | RT | p(corr) | VIP | Delta(ppm) | MS/MS fragment ion (m/z) | Level of Assignment |
| --- | --- | --- | --- | --- | --- | --- | --- | --- | --- |
|  | **KKU-100** |  |  |  |  |  |  |  |  |
| 1 | *S*-Acetyldihydrolipoamide | [M-H]^-^ | 248.95 | 1.09 | 0.80 | 1.11 | 0.87 | - | 1 |
| 2 | Methylselenopyruvate | [M-H]^-^ | 180.96 | 1.13 | 0.84 | 1.23 | 105 | - | 1 |
| 3 | TG (60:11) | [M+NH_4_]^+^ | 970.78 | 15.65 | 0.85 | 3.27 | 0.31 | 201.16; 623.50 | 2 |
| 4 | TG (56:8) | [M+NH_4_]^+^ | 920.77 | 15.67 | 0.80 | 2.65 | 0.18 | 201.16; 647.51 | 2 |
| 5 | TG (58:9) | [M+NH_4_]^+^ | 946.78 | 15.76 | 0.82 | 2.51 | 1.03 | 203.18; 601.52 | 2 |
| 6 | TG (50:4) | [M+NH_4_]^+^ | 844.74 | 15.76 | 0.87 | 2.54 | 1.1 | - | 1 |
| 7 | TG (60:10) | [M+NH_4_]^+^ | 972.80 | 15.79 | 0.87 | 3.50 | 0.12 | 265.25; 625.52 | 2 |
| 8 | TG (52:5) | [M+H]^+^ | 870.75 | 15.81 | 0.85 | 2.66 | 1.47 | - | 1 |
| 9 | TG (56:7) | [M+H]^+^ | 922.78 | 15.92 | 0.61 | 3.61 | 2.44 | - | 1 |
| 10 | TG (50:3) | [M+NH_4_]^+^ | 846.75 | 16.02 | 0.89 | 5.72 | 1.96 | - | 1 |
| 11 | TG (57:7) | [M+NH_4_]^+^ | 936.80 | 16.03 | 0.87 | 2.03 | 2.87 | - | 1 |
| 12 | TG (48:2) | [M+NH_4_]^+^ | 820.74 | 16.03 | 0.91 | 4.48 | 0.98 | - | 1 |
| 13 | TG (55:6) | [M+H]^+^ | 910.78 | 16.05 | 0.86 | 2.88 | 0.55 | - | 1 |
| 14 | TG (54:5) | [M+NH_4_]^+^ | 898.78 | 16.05 | 0.88 | 4.97 | 1.59 | - | 1 |
| 15 | TG (52:4) | [M+NH_4_]^+^ | 872.77 | 16.06 | 0.88 | 5.70 | 1.65 | 261.22; 575.51 | 2 |
| 16 | TG (46:7) | [M+NH_4_]^+^ | 922.78 | 16.08 | 0.89 | 6.13 | 2.44 | 313.26; 623.51; 905.76 | 2 |
| 17 | TG (53:4) | [M+NH_4_]^+^ | 886.78 | 16.17 | 0.88 | 2.87 | 2.75 | - | 1 |
| 18 | TG (51:3) | [M+NH_4_]^+^ | 860.77 | 16.18 | 0.88 | 3.60 | 2.99 | - | 1 |
| 19 | TG (58:7) | [M+NH_4_]^+^ | 950.81 | 16.18 | 0.91 | 5.24 | 3.73 | 339.29; 651.54; 933.794 | 2 |
| 20 | TG (49:2) | [M+H]^+^ | 834.75 | 16.19 | 0.87 | 2.92 | 1.65 | - | 1 |
| 21 | TG (60:8) | [M+H]^+^ | 976.83 | 16.19 | 0.89 | 1.99 | 3.29 | - | 1 |
| 22 | TG (56:6) | [M+H]^+^ | 924.80 | 16.2 | 0.88 | 8.57 | 2.81 | - | 1 |
| 23 | TG (54:4) | [M+NH_4_]^+^ | 900.80 | 16.33 | 0.89 | 6.57 | 3.48 | 263.24; 601.52 | 2 |
| 24 | TG (48:1) | [M+NH_4_]^+^ | 822.75 | 16.33 | 0.90 | 4.69 | 1.35 | - | 1 |
| 25 | TG (52:3) | [M+NH_4_]^+^ | 874.78 | 16.33 | 0.91 | 8.83 | 1.77 | 263.24; 575.51 | 2 |
| 26 | TG (50:2) | [M+NH_4_]^+^ | 848.77 | 16.34 | 0.91 | 6.85 | 1.65 | 311.26; 549.49 | 2 |
| 27 | TG (56:5) | [M+H]^+^ | 926.81 | 16.4 | 0.86 | 4.58 | 0.67 | 289.25; 577.52 | 2 |
| 28 | TG (53:3) | [M+NH_4_]^+^ | 888.80 | 16.45 | 0.88 | 3.55 | 2.62 | 313.28; 589.52 | 2 |
| 29 | TG (51:2) | [M+NH_4_]^+^ | 862.78 | 16.46 | 0.86 | 4.89 | 1.35 | - | 1 |
| 30 | TG (49:1) | [M+NH_4_]^+^ | 836.77 | 16.48 | 0.84 | 3.36 | 0.73 | - | 1 |
| 31 | TG (58:6) | [M+NH_4_]^+^ | 952.83 | 16.5 | 0.87 | 3.10 | 2.32 | - | 1 |
| 32 | TG (54:3) | [M+H]^+^ | 902.81 | 16.59 | 0.90 | 6.34 | 1.77 | - | 1 |
| 33 | TG (52:2) | [M+NH_4_]^+^ | 876.80 | 16.6 | 0.88 | 9.55 | 0.85 | - | 1 |
| 34 | TG (56:4) | [M+H]^+^ | 928.83 | 16.61 | 0.91 | 3.08 | 3.35 | 265.25; 605.55 | 2 |
| 35 | TG (53:2) | [M+H]^+^ | 890.81 | 16.74 | 0.83 | 3.32 | 1.65 | 95.09; 577.52 | 2 |
|  | **KKU-213A** |  |  |  |  |  |  |  |  |
| 1 | Sphinganine | [M+H]^+^ | 274.27 | 1.01 | 0.62 | 2.42 | 0.00 | 88.07; 321.25; 274.27 | 2 |
| 2 | PI (20:4/0:0) | [M+NH_4_]^+^ | 638.34 | 3.38 | 0.76 | 1.84 | 9.00 | - | 1 |
| 4 | TG (62:12) | [M+NH_4_]^+^ | 996.80 | 15.64 | 0.99 | 2.10 | 2.68 | - | 1 |
| 4 | TG (60:11) | [M+NH_4_]^+^ | 970.78 | 15.65 | 1.00 | 2.58 | 0.31 | 201.16; 623.50 | 2 |
| 5 | TG (56:8) | [M+NH_4_]^+^ | 920.77 | 15.67 | 0.99 | 3.07 | 0.18 | 201.16; 647.51 | 2 |
| 6 | TG (54:7) | [M+NH_4_]^+^ | 896.77 | 15.75 | 0.99 | 3.10 | 2.81 | 311.26; 597.49 | 2 |
| 7 | TG (58:9) | [M+NH_4_]^+^ | 946.78 | 15.76 | 0.99 | 4.52 | 1.03 | 203.18; 601.52 | 2 |
| 8 | TG (50:4) | [M+NH_4_]^+^ | 844.74 | 15.76 | 0.99 | 1.92 | 1.1 | - | 1 |
| 9 | TG (60:10) | [M+NH_4_]^+^ | 972.80 | 15.79 | 0.99 | 2.66 | 0.12 | 265.25; 625.52 | 2 |
| 10 | TG (52:5) | [M+H]^+^ | 870.75 | 15.81 | 0.99 | 2.43 | 1.47 | - | 1 |
| 11 | TG (58:8) | [M+H]^+^ | 953.75 | 15.91 | 0.98 | 1.95 | 3.24 | - | 1 |
| 12 | TG (56:7) | [M+H]^+^ | 922.78 | 15.92 | 1.00 | 5.52 | 2.44 | - | 1 |
| 13 | TG (50:3) | [M+NH_4_]^+^ | 846.75 | 16.02 | 0.99 | 4.18 | 1.96 | - | 1 |
| 14 | TG (48:2) | [M+NH_4_]^+^ | 820.74 | 16.03 | 0.99 | 2.75 | 0.98 | - | 1 |
| 15 | TG (57:7) | [M+NH_4_]^+^ | 936.80 | 16.03 | 0.98 | 1.71 | 2.87 | - | 1 |
| 16 | TG (55:7) | [M+H]^+^ | 910.78 | 16.05 | 0.99 | 2.26 | 0.55 | - | 1 |
| 17 | TG (54:5) | [M+N_H4_]^+^ | 898.78 | 16.05 | 0.99 | 4.95 | 1.59 | - | 1 |
| 18 | TG (52:4) | [M+NH_4_]^+^ | 872.77 | 16.06 | 0.99 | 5.09 | 1.65 | 261.22; 575.51 | 2 |
| 19 | TG (56:4) | [M+NH_4_]^+^ | 922.78 | 16.08 | 0.99 | 5.89 | 2.44 | 313.26; 623.51; 905.76 | 2 |
| 20 | TG (53:4) | [M+NH_4_]^+^ | 886.78 | 16.17 | 0.99 | 2.69 | 2.75 | - | 1 |
| 21 | TG (58:7) | [M+Na]^+^ | 955.77 | 16.18 | 0.97 | 1.84 | 0.43 | - | 1 |
| 22 | TG (58:7) | [M+NH_4_]^+^ | 950.81 | 16.18 | 0.98 | 4.95 | 3.73 | 339.29; 651.54; 933.794 | 2 |
| 23 | TG (51:3) | [M+NH_4_]^+^ | 860.77 | 16.18 | 0.99 | 2.89 | 2.99 | - | 1 |
| 24 | TG (60:8) | [M+H]^+^ | 976.83 | 16.19 | 0.99 | 1.83 | 3.29 | - | 1 |
| 25 | TG (49:2) | [M+H]^+^ | 834.75 | 16.19 | 0.99 | 1.80 | 1.65 | - | 1 |
| 26 | TG (56:6) | [M+H]^+^ | 924.80 | 16.2 | 0.99 | 6.97 | 2.81 | - | 1 |
| 27 | TG (54:4) | [M+NH_4_]^+^ | 900.80 | 16.33 | 0.98 | 6.63 | 3.48 | 263.24; 601.52 | 2 |
| 28 | TG (52:3) | [M+NH_4_]^+^ | 874.78 | 16.33 | 0.98 | 7.99 | 1.77 | 263.24; 575.51 | 2 |
| 29 | TG (48:1) | [M+NH_4_]^+^ | 822.75 | 16.33 | 0.70 | 2.03 | 1.35 | - | 1 |
| 30 | TG (52:3) | [M+Na]^+^ | 879.74 | 16.34 | 0.98 | 2.26 | 1.46 | - | 1 |
| 31 | TG (54:4) | [M+Na]^+^ | 905.75 | 16.34 | 0.95 | 1.83 | 1.9 | - | 1 |
| 32 | TG (50:2) | [M+NH_4_]^+^ | 848.77 | 16.34 | 0.97 | 4.80 | 1.65 | 311.26; 549.49 | 2 |
| 33 | TG (57:6) | [M+Na]^+^ | 938.81 | 16.34 | 0.99 | 1.73 | 4.03 | - | 1 |
| 34 | TG (56:5) | [M+H]^+^ | 926.81 | 16.4 | 0.99 | 3.50 | 0.67 | 289.25; 577.52 | 2 |
| 35 | TG (53:3) | [M+NH_4_]^+^ | 888.80 | 16.45 | 0.96 | 2.68 | 2.62 | 313.28; 589.52 | 2 |
| 36 | TG (51:2) | [M+NH_4_]^+^ | 862.78 | 16.46 | 0.93 | 2.95 | 1.35 | - | 1 |
| 37 | TG (49:1) | [M+NH_4_]^+^ | 836.77 | 16.48 | 0.90 | 1.89 | 0.73 | - | 1 |
| 38 | TG (58:60) | [M+NH_4_]^+^ | 952.83 | 16.5 | 0.99 | 4.07 | 2.32 | - | 1 |
| 39 | TG (54:3) | [M+H]^+^ | 902.81 | 16.59 | 0.84 | 4.30 | 1.77 | - | 1 |
| 40 | TG (52:2) | [M+NH_4_]^+^ | 876.80 | 16.6 | 0.69 | 4.83 | 0.85 | - | 1 |
| 41 | TG (56:4) | [M+H]^+^ | 928.83 | 16.61 | 0.96 | 2.59 | 3.35 | 265.25; 605.55 | 2 |
| 42 | TG (58:5) | [M+NH_4_]^+^ | 954.84 | 16.7 | 0.99 | 2.06 | 4.15 | - | 1 |
| 43 | TG (53:2) | [M+H]^+^ | 890.81 | 16.74 | 0.67 | 1.91 | 1.65 | 95.09; 577.52 | 2 |

PI, phosphatidylinositol; TG, triglyceride; m/z, observed mass-to-charge ratio; RT, retention time (min).

Level of Assignment; 1: Tentative assignment; 2: Tandem MS spectrum matched to database or literature.
