# Supplementary material for: Lipidomic Analyses Uncover Apoptotic and Inhibitory Effects of Pyrvinium Pamoate on Cholangiocarcinoma Cells via Mitochondrial Membrane Potential Dysfunction
Source: Front Public Health. 2021 Dec 7;9:766455. doi: 10.3389/fpubh.2021.766455 (PMC8688698; doi:10.3389/fpubh.2021.766455)
Supplement: Supplementary Table 2 — The candidate lipid species identification of KKU-100 and KKU-213 cells associated with mitochondrial membrane potential after treatment with PP, related to Figure 6. [file Table_2.DOCX]

Supplementary Material

**Table S2.** The candidate lipid species identification of KKU-100 and KKU-213 cells associated with mitochondrial membrane potential after treatment with PP, related to Figure 6.

| **No.** | **Lipid molecular species** | **Ion species** | **m/z** | **RT** | **p(corr)** | **VIP (ATP)** | **VIP (TMRE)** | **Delta (ppm)** | **MS/MS fragment ion (m/z)** | **Level of Assignment** |
| --- | --- | --- | --- | --- | --- | --- | --- | --- | --- | --- |
| 1 | TG (52:2) | [M+NH_4_]^+^ | 876.80 | 16.60 | 0.91 | 9.54 | 9.54 | 1.59 | 265.25; 577.52; 841.78 | 2 |
| 2 | TG (52:3) | [M+NH_4_]^+^ | 874.78 | 16.33 | 0.94 | 8.78 | 8.81 | 2.07 | 263.24; 575.51 | 2 |
| 3 | TG (56:6) | [M+NH_4_]^+^ | 924.80 | 16.20 | 0.92 | 8.57 | 8.56 | 1.71 | 313.26; 578.52 | 2 |
| 4 | TG (50:2) | [M+NH_4_]^+^ | 848.77 | 16.34 | 0.93 | 6.80 | 6.82 | 2.32 | 311.26; 549.49 | 2 |
| 5 | TG (54:4) | [M+NH_4_]^+^ | 900.80 | 16.33 | 0.92 | 6.57 | 6.57 | 1.53 | 263.24; 601.52 | 2 |
| 6 | TG (54:3) | [M+NH_4_]^+^ | 902.81 | 16.59 | 0.93 | 6.30 | 6.32 | 1.28 | 265.25; 603.54; 902.83 | 2 |
| 7 | TG (56:7) | [M+NH_4_]^+^ | 922.78 | 16.08 | 0.92 | 6.11 | 6.11 | 0.36 | 313.26; 623.51 | 2 |
| 8 | TG (50:3) | [M+NH_4_]^+^ | 846.75 | 16.02 | 0.92 | 5.69 | 5.71 | 0.55 | 311.26; 547.47 | 2 |
| 9 | TG (52:4) | [M+NH_4_]^+^ | 872.77 | 16.06 | 0.91 | 5.69 | 5.69 | 0.49 | 261.22; 575.05 | 2 |
| 10 | TG (58:7) | [M+NH_4_]^+^ | 950.81 | 16.18 | 0.94 | 5.21 | 5.22 | 0.92 | 339.29; 651.54; 933.79 | 2 |

TG, triglyceride; m/z, observed mass-to-charge ratio; RT, retention time (min)

Level of Assignment; 1: Tentative assignment; 2: Tandem MS spectrum matched to database or literature.
